# Impact of autologous platelet-rich plasma therapy vs. hyaluronic acid on synovial fluid biomarkers in knee osteoarthritis: a randomized controlled clinical trial

**DOI:** 10.3389/fmed.2023.1258727

**Published:** 2023-10-05

**Authors:** Tianshu Li, Yuefang Li, Wanyue Li, Xu Wang, Qixin Ding, Jiahuan Gao, Ying Zhang, Weisheng Zhuang

**Affiliations:** ^1^Department of Rehabilitation Medicine, People’s Hospital of Henan University, The First People’s Hospital of Zhengzhou, Zhengzhou, Henan, China; ^2^Department of Rehabilitation, The First Affiliated Hospital of Jinan University, Guangzhou, Guangdong, China; ^3^School of Rehabilitation Medicine, Henan University of Chinese Medicine, Zhengzhou, China; ^4^Department of Rehabilitation Medicine, Henan Provincial People’s Hospital, People’s Hospital of Zhengzhou University, Zhengzhou, Henan, China

**Keywords:** knee osteoarthritis, platelet-rich plasma, biomarkers, inflammatory cytokines, articular cavity injection

## Abstract

**Objective:**

Observe the effects of platelet-rich plasma (PRP) therapy on inflammatory cytokines in the synovial fluid of the knee joint of patients with KOA, and explore the effects of PRP intra-articular injection on the inflammation of the knee joint environment and the possible mechanism of action.

**Methods:**

Seventy patients were randomized to undergo three blinded weekly intra-articular injections of PRP or hyaluronic acid (HA). The concentrations of inflammatory cytokines, including interleukin (IL)-6, IL-1β, tumor necrosis factor (TNF)-α, IL-8, IL-17A, IL-17F, IL-4, IL-5, and IL-10, in the synovial fluid were evaluated before the intervention and 1 month after the third injection. The Western Ontario and McMaster University (WOMAC) and visual analog scale (VAS) scores were used to assess pain and functional status of the knee joints in both groups before the intervention, immediately post-intervention, and 1, 3, 6, and 12 months after the intervention.

**Results:**

Baseline characteristics were similar in both groups with no statistical difference. The IL-6, IL-1β, TNF-α, IL-17A, and IL-10 levels in the synovial fluid of the observation group decreased significantly after, vs. before, the intervention (*p* < 0.05), whereas the IL-8, IL-17F, and IL-4 levels decreased (*p* > 0.05) and IL-5 levels increased (*p* > 0.05). There was no statistically significant difference between inflammatory cytokine levels in the synovial fluid of the samples from the control group before and after the intervention (*p* > 0.05). There were no statistically significant differences between the two groups immediately after intervention (*p* > 0.05). At 1, 3, 6, and 12 months after intervention, the WOMAC and VAS scores were significantly better in the observation group than in the control group (*p* < 0.05).

**Conclusion:**

Platelet plasma therapy can reduce the concentrations of inflammatory cytokines IL-6, IL-1β, TNF-α, IL-17A, and IL-10 in the synovial fluid of KOA patients, reduce the expression levels of IL-8, IL-17F, and IL-4, clear the pro-inflammatory factors, improve the inflammatory environment of the affected knee joint, and alleviate pain caused by inflammation. Thus, alleviating pain and improving knee function in patients with KOA.

## 1. Introduction

Knee Osteoarthritis (KOA) is a degenerative joint disease characterized by pain, swelling, stiffness, and dysfunction (1). Pain and limited joint function significantly affect the quality of life of the elderly and impose a large economic burden on their families and society (2). The pathogenesis of KOA is complex and includes degenerative changes in the articular cartilage and subchondral bone, acute and chronic synovial inflammation, and decreased synovial viscosity, all of which lead to joint structure destruction and synovial injury (3, 4).

For patients with knee OA who cannot tolerate oral medication treatment, intra-articular injection is recommended as an alternative treatment option to delay surgical intervention (5–7). Hyaluronic Acid (HA), a synthesized product, has a controversial role in cartilage repair and delaying disease progression (8). Platelet-Rich Plasma (PRP), obtained by centrifuging whole blood to concentrate platelets and plasma, releases a large number of growth factors and cytokines after activation through various pathways that play an important role in tissue repair (9). PRP exerts anti-inflammatory and pain-relieving effects by inhibiting the expression of inflammatory cytokines in the synovial and cartilaginous tissues. The growth factors released by PRP promote cell aggregation, proliferation, and angiogenesis; thereby reducing key regulatory factors in the inflammatory process and decreasing the expression of inflammatory enzymes, such as interleukin-1β (IL-1β), tumor necrosis factor-α (TNF-α), and matrix metalloproteinases (MMPs) (8, 10).

Currently, it is believed that the development and progression of KOA during its pathogenesis, even in the early stages of the disease, involves inflammation (11). Studies have demonstrated a clear link between the progression of tibial cartilage injury and synovial inflammation (12, 13). This has led to increasing attention on biological factors, such as cytokines, affecting the synovial fluid and articular cartilage, and cytokines have become an important focus of research on the treatment and prevention of KOA (14). The identification of OA- and joint-specific biomarkers for the early diagnosis of OA is useful for the development of prevention and treatment programs (15). An in-depth study of biomarkers can provide opportunities for better diagnosis and disease stratification (16). Through the measurement of biomarkers, we can deepen our understanding of the pathogenesis of knee osteoarthritis and the mechanism of action of different treatment schemes, and also help to observe the impact of PRP injection therapy on the joint environment and inflammation state. This study aimed to observe the effects of PRP and HA therapy on inflammatory cytokines in the synovial fluid of the knee joint of patients with KOA and the pain and function of the knee joint. Moreover, we aimed to set inflammatory cytokines as the main observation indicators to explore the effects of PRP intra-articular injection on the inflammation of the knee joint environment and the possible mechanism of action.

## 2. Patients and methods

In this single-blind, prospective, randomized study, 70 patients with knee osteoarthritis who met the inclusion and exclusion criteria and received outpatient and inpatient treatment at Henan Provincial People’s Hospital from January 2021 to October 2022 were included. Patients were divided into an observation group (35 cases) and a control group (35 cases) according to the random number table method. The study protocol was thoroughly reviewed and approved by the appropriate ethics committee (Ethical Lot Number: 2018057). The study protocol was registered with the China Clinical Trials Registry (Clinical Registration Number: ChiCTR2100043259) to ensure transparency and accountability.

**Figure 1:**
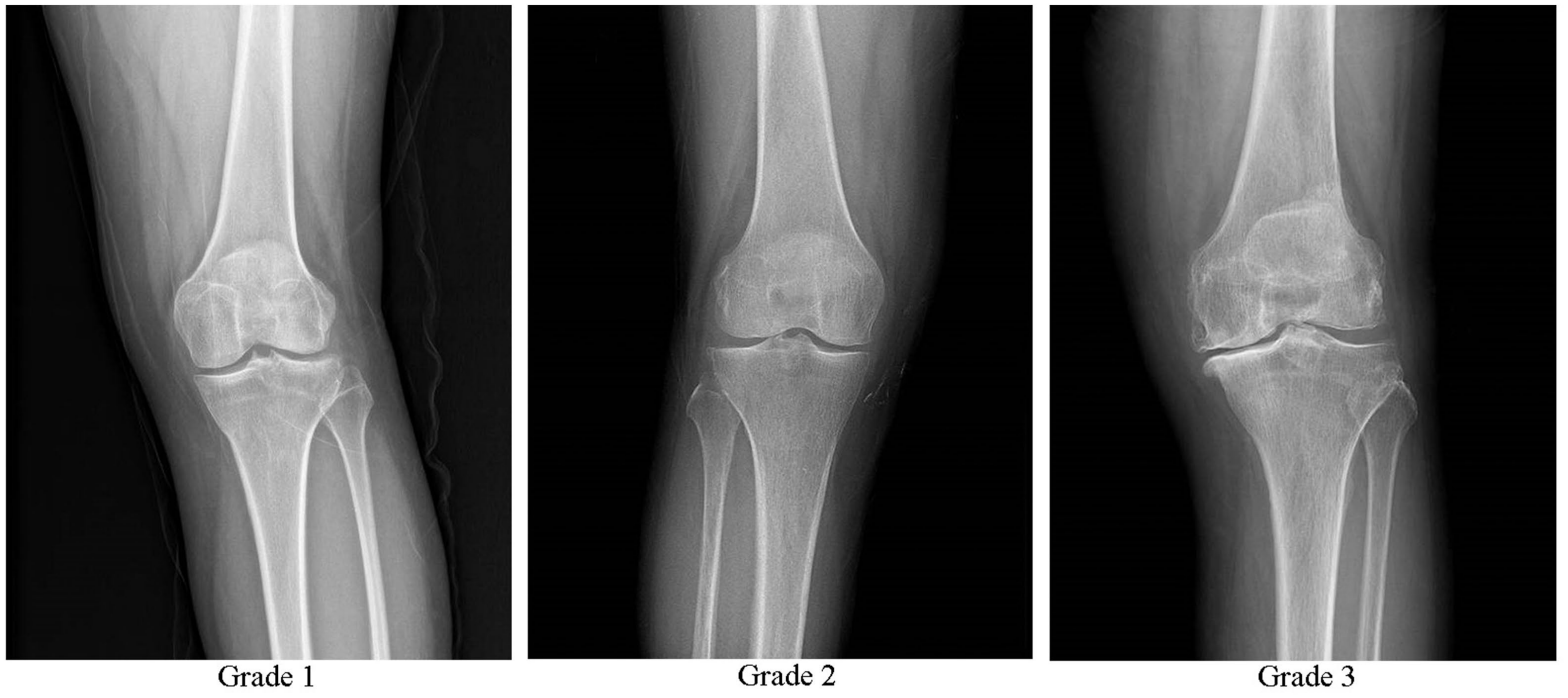
Osteoarthritis of the knee Kellgren-Lawrence grade 1, 2, and 3.

Inclusion criteria included meeting the diagnostic criteria for KOA in the Osteoarthritis Diagnosis and Treatment Guidelines (2018 Edition) (17): age ≥ 35 to ≤75 years, chronic joint pain or swelling (> 4 months), and X-ray results meeting the Kellgren-Lawrence scale (K-L scale) level I–III (Figure 1) (18, 19). The presence of intra-articular effusion (hydrops) with the suprapatellar recess distension of >4 mm was verified by the ultrasound. In addition, the patient’s had a desire for treatment and good family support. The patients were informed about the study and provided written informed consent. The exclusion criteria included complicated rheumatoid arthritis, severe osteoporosis, tumor, gout, diabetes, blood disease, severe cardiovascular disease, infection, previous use of anticoagulants, immunosuppressants, and non-steroidal anti-inflammatory drugs within 5 days, recent history of knee joint surgery, platelet count <150 × 10^9^ /L, severe coagulation disorders, injection site skin ulcers, significant joint deformities (knee joint varus or valgus >5°), pregnancy, and lactation.

### 2.1. PRP preparations and characteristics

Platelet-rich plasma was prepared using a two-step centrifugation method (20). Under sterile conditions, 2 mL of sodium citrate anticoagulant (Batch No: 190228266, Sichuan Nangya Biotechnology Co., Ltd.) was extracted, followed by collection of 18 mL of venous blood from the median cubital vein of the patient, totaling 20 mL. After thorough mixing, the blood was placed in a low-speed centrifuge (Model H1850, Hunan Xiangyi Centrifuge Instrument Co., Ltd.) for centrifugation. The first centrifugation step involved applying a relative centrifugal force of 200 *g* for 10 min. After the first centrifugation, whole blood was separated into three layers. The supernatant, intermediate layer, and layer approximately 3 mm below the interface were collected for a second centrifugation step. The second centrifugation step involved applying a relative centrifugal force of 200 *g* for 20 min. After centrifugation, approximately three-fourths of the lower platelet-poor plasma was removed, leaving approximately 4 mL of PRP, which was extracted using a 5 mL sterile syringe and stored for later use. In the PRP prepared using this method, the platelet concentration is approximately four times higher than the baseline level, while the average leukocyte concentration is 2.5–2.9-fold higher than that in normal blood, making it leukocyte-rich PRP.

Blood and PRP samples were randomly and regularly collected from patients receiving treatment. Both types of samples were analyzed using a blood analyzer (Sysmex XN-9100, Sysmex Corporation, Japan) to verify the correct preparation of PRP and ensure compliance with manufacturer specifications. The initial platelet concentration was 209.25 ± 32.99 × 10^9^/L. The average concentration of PRP obtained from blood samples was 826.37 ± 41.26 × 10^9^/L, which contained leukocytes and a small amount of red blood cells. Therefore, in accordance with the PRP classification and coding system proposed by Kon (21), the PRP code used in this study was 28-11-00. The specific characteristics of the PRP classifications are presented in Table 1.

**Table 1:** Classification features of PRP.

Parameter		Values
PRP preparation		
Initial blood volume		18 mL
Anticoagulant		2.5% sodium citrate 2 mL
System		Close
Centrifugation		Yes
Number		2
First centrifugal force		200 × g for 10 min
Second centrifugal force		200 × g for 20 min
Final PRP volume		4 mL
PRP characteristics		
PRP type		28–11-00
Initial blood platelet concentration		209.25 ± 32.99(10^9^/L)
Platelet concentration in PRP		826.37 ± 41.26(10^9^/L)
Does it contain red blood cells? (0 = No, 1 = Yes)		1
Does it contain white blood cells? (0 = No, 1 = Yes)		1
Initial leukocyte concentration		5.82 ± 1.63(10^9^/L)
Leukocyte concentration in PRP		15.80 ± 3.25(10^9^/L)
Mode of activation (0 = Endogenous activation, 1 = Activation prior to injection.)		0
Calcium agent used for activation(0 = No, 1 = Yes)		0
Application characteristics		
Formulation type		Liquid
Administration route		Intraarticular or intraosseous
Dosage		A treatment session is conducted once a week, totaling three sessions
Volume		Intraarticular injection: 4 mL
Dose (range of platelets)		Intraarticular injection:2.75 × 109–3.03 × 10^9^
Tissue		Cartilage, synovium, and subchondral bone
Pathology		Knee joint degeneration

### 2.2. Treatment and evaluation

Both groups received health education and quadriceps training. Quadriceps training began 24 h after injection therapy and was guided by a therapist. Before each treatment session, preparatory activities consisting mainly of active limb movements and knee flexion and extension exercises were performed for 3–5 min. The quadriceps training methods included non-weight-bearing straight leg raises, static squats, and isometric exercises. These training sessions were conducted three times per week for 6 consecutive weeks.

**Figure 2:**
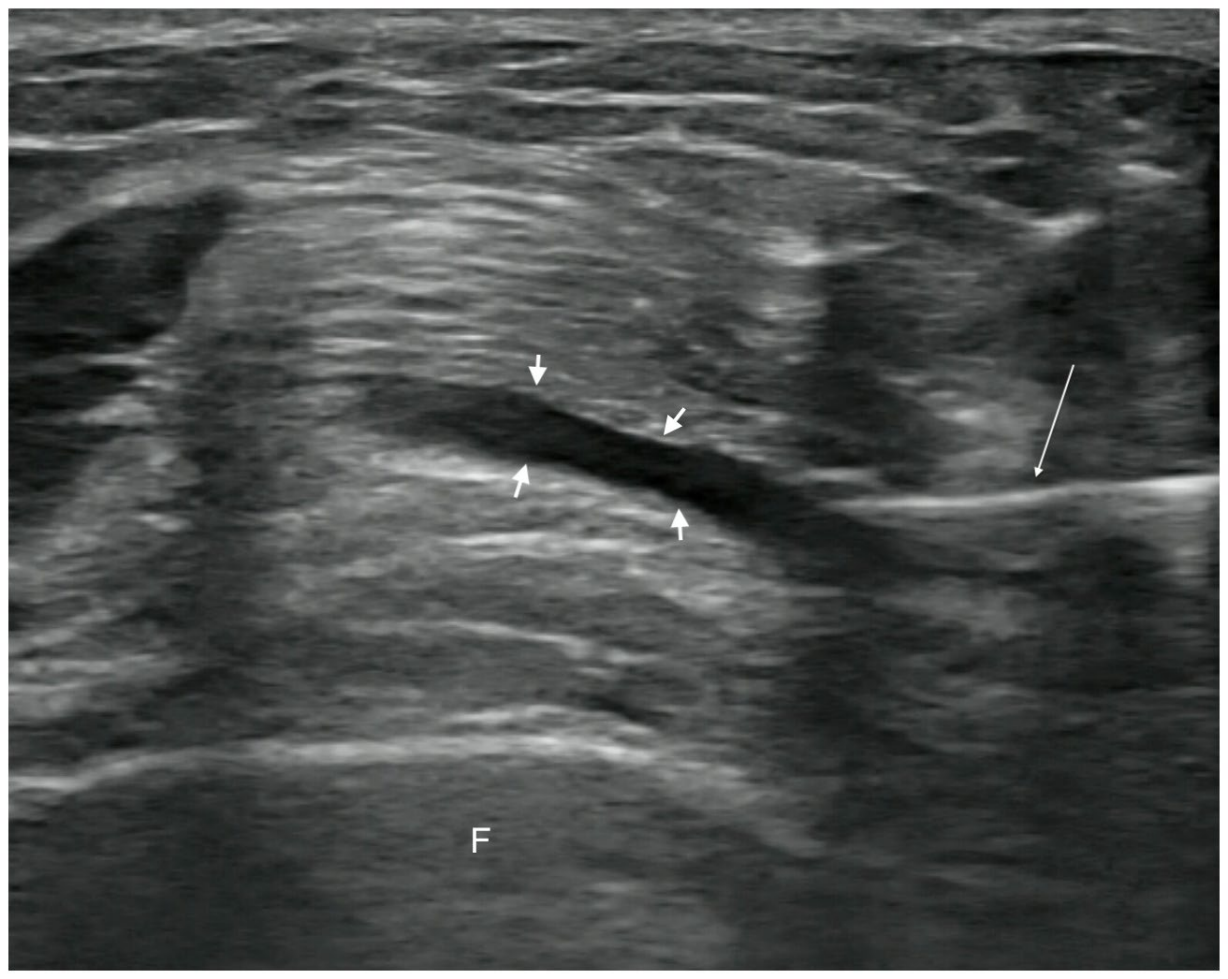
Pre-injection image. Short arrow, suprapatellar bursa. Long arrow, syringe needle. F, femur.

**Figure 3:**
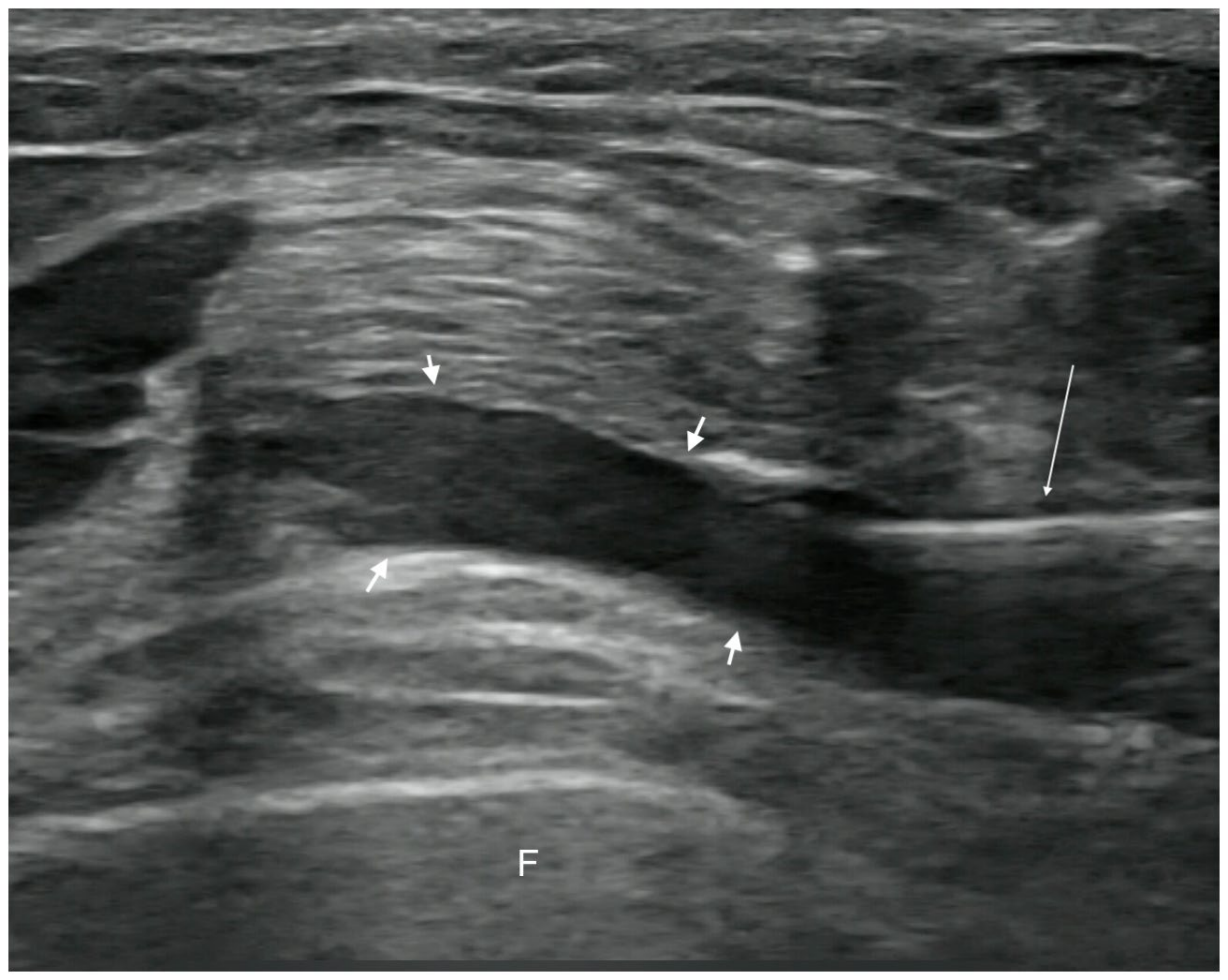
Post-injection image.

In the observation group, patients received intra-articular injections of PRP. Injections were administered with the patient in the supine position, the affected knee slightly flexed, and the injection site thoroughly exposed. Strict disinfection protocols were followed, and a sterile coupling agent (Guangdong brand, approved by Yue-Sui Medical Device No. 20160194) was used. An experienced physician injected the patient under ultrasound guidance (Sonimage HS1 Plus; Konica Minolta, Inc.), targeting the suprapatellar bursa of the affected knee. After completing the treatment, pressure was applied using a cotton swab to stop the bleeding, and a sterile dressing was applied to cover the injection site. Images of the ultrasound-guided intra-articular injections are shown in Figures 2, 3. Passive flexion and extension exercises of the affected knee were performed 3–5 times before discharge, and the patients were observed for 20 min without any abnormal signs. Each injection consisted of 4 mL of PRP, administered once a week for 3 consecutive weeks.

In the control group, sodium hyaluronate injection (Shandong Bausch & Lomb Freda Pharmaceutical Co., Ltd.; National Medical Products Administration Approval No. H10960136) was administered intra-articularly following the same injection protocol as the observation group. Each injection consisted of 2 mL aliquots containing 20 mg of sodium hyaluronate and was administered once a week for 3 consecutive weeks. The average molecular weight was 0.7–1.4 × 10^6^ Da.

Post-treatment management involved instructing patients to rest for 24 h and avoid contact with water at the injection site within the first 3 days to prevent infection. Patients were advised to avoid excessive weight-bearing, walking, and vigorous activities for 1 week. Follow-up assessments were conducted, and patients were prohibited from using any medication other than baseline treatment during the study period. Prompt medical intervention was recommended if discomfort occurred.

### 2.3. Biochemical assay

The concentrations of inflammatory cytokines, including IL-6, IL-1β, TNF-α, IL-8, IL-17A, IL-17F, IL-4, IL-5, and IL-10, in the synovial fluid were evaluated before intervention and 1 month after the third injection. IL-6, IL-1β, and TNF-α were considered key pro-inflammatory cytokines, while IL-8, IL-17A, IL-17F, IL-4, IL-5, and IL-10 were commonly observed inflammatory cytokines. The specific procedure was as follows: approximately 2 mL of synovial fluid was extracted before the first injection and 1 week after the third injection under ultrasound guidance. The samples were stored at −80°C and cytokine concentrations were measured using a multiplex microsphere flow cytometry-based assay (Qingdao Rui-sky Cell Cytokine Detection Kit, Q.L. Medical Device No. 20180087).

### 2.4. Patient-reported outcomes

**Figure 4:**
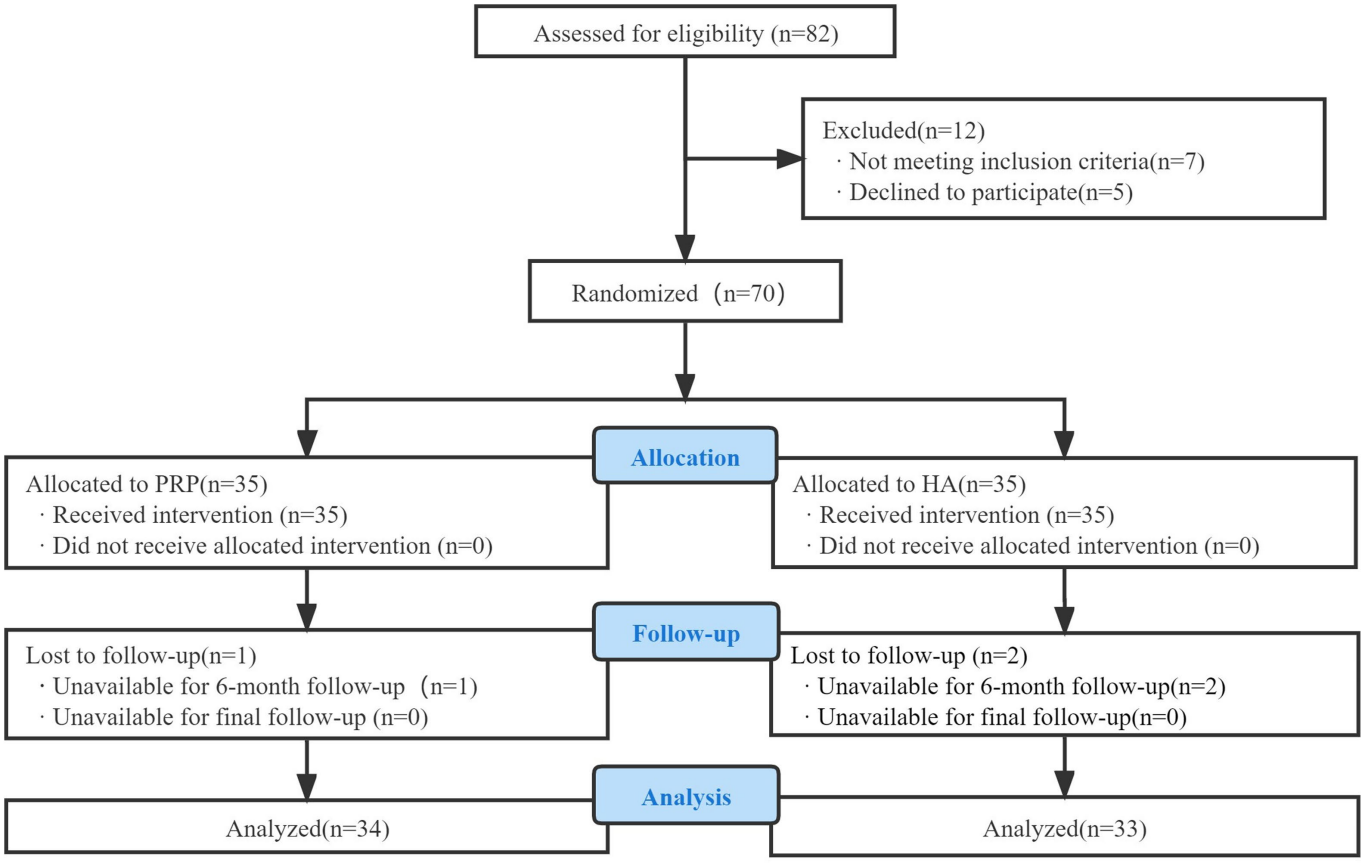
Consolidated standards of reporting trials (CONSORT) flow diagram used in the design of the trial.

The Western Ontario and McMaster University (WOMAC) (22, 23) and Osteoarthritis Index and Visual Analog Scale (VAS) (24) were used to assess pain and functional status of the knee joints in both groups before the intervention, immediately post-intervention, and 1, 3, 6, and 12 months after the intervention. A flowchart of the study is shown in Figure 4.

### 2.5. Statistical analysis

Data analysis was performed using IBM SPSS Statistics version 26.0 (IBM Corp., Armonk, NY, United States). Measurement data were presented as mean ± standard deviation (x̅ ± s), while count data were presented as frequencies. Independent sample *t*-tests were used to compare normally distributed baseline measurement data between the groups, while chi-square tests were used to compare count data. Two-factor repeated-measures ANOVA was employed to compare the WOMAC, AKS, and VAS scores. Student’s *t*-test or rank-sum test was used to compare cytokine concentrations. Statistical significance was set at *p* < 0.05.

## 3. Results

Among the 70 enrolled patients, one in the observation group and two in the control group were lost to follow-up. Thus, 67 patients (21 males and 46 females) were included in this study. Their ages ranged from 35 to 75 years, with a mean age of 59.22 ± 9.09 years. Statistical analysis revealed no significant differences (*p* > 0.05) between the two groups in terms of sex, age, K-L scale, or BMI, indicating comparability. The baseline characteristics of the patient population were similar between the groups. Specific data are listed in Table 2.

**Table 2:** Participant characteristics.

Parameters	Participants, No. or Mean ± SD		
	PRP (*n* = 34)	HA (*n* = 33)	*p* value
Sex, male: female	8:26	13:20	0.162
Age, years	59.53 ± 8.19	58.91 ± 10.06	0.783
BMI, kg/m^2^	25.56 ± 1.94	25.83 ± 2.28	0.604
K-L classification			0.893
Grade 1	14	14	
Grade 2	12	10	
Grade 3	8	9	

### 3.1. Cellular cytokine analysis

Among the 67 patients in this study, at 1 month after the third injection, nine patients in the observation group and eight patients in the control group were excluded because of the inability to collect synovial fluid or the presence of blood contamination in the synovial fluid prior to PRP injection. Finally, synovial fluid was obtained from 50 patients pre- and post-intervention for the comparison of cytokines. Please refer to Table 3 for the specific missing data.

**Table 3:** The absence of synovial fluid in the patient groups at 1 month after the third injection.

Group	The inclusion of the study sample size (*n*)	Missing values (*n*)	Causes of missing values	The final sample size for cytokines comparison (*n*)
Observation	34	9	Eight individuals with unobtainable synovial fluid/one individual with synovial fluid contamination by blood.	25
Control	33	8	Seven individuals with unobtainable synovial fluid/one individual with synovial fluid contamination by blood.	25

**Figure 5:**
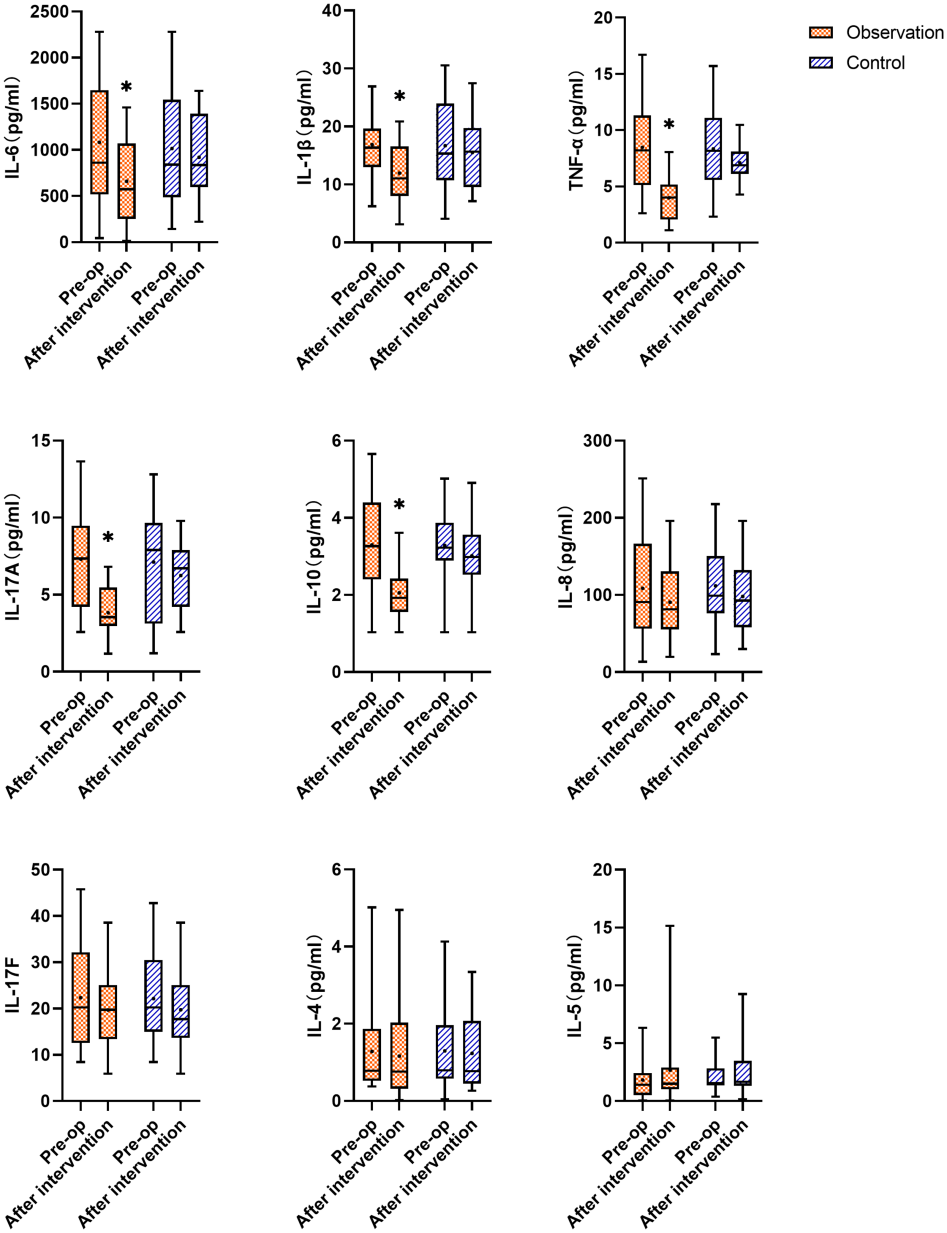
Changes in cytokines IL-6, IL-1β, TNF-α, IL-17A, IL-10, IL-8, IL-17F, IL-4, and IL-5 in the synovial fluid of the observation and the control groups before and after the intervention; ^*^ represents statistically significant differences before and after treatment (*p* < 0.05).

As shown in Figure 5, there was no statistically significant difference in cytokine levels between the two groups before the intervention (*p* > 0.05). After the intervention, the levels of IL-6, IL-1β, TNF-α, IL-17A, and IL-10 in the synovial fluid of the observation group decreased significantly compared to before the intervention (*p* < 0.05), whereas the levels of IL-8, IL-17F, and IL-4 decreased (*p* > 0.05), and the level of IL-5 increased (*p* > 0.05). There was no statistically significant difference in the levels of IL-6, IL-1β, TNF-α, IL-17A, IL-10, IL-8, IL-17F, IL-4, and IL-5 in the synovial fluid of the control group after the intervention compared to before treatment (*p* > 0.05).

### 3.2. Clinical results

**Figure 6:**
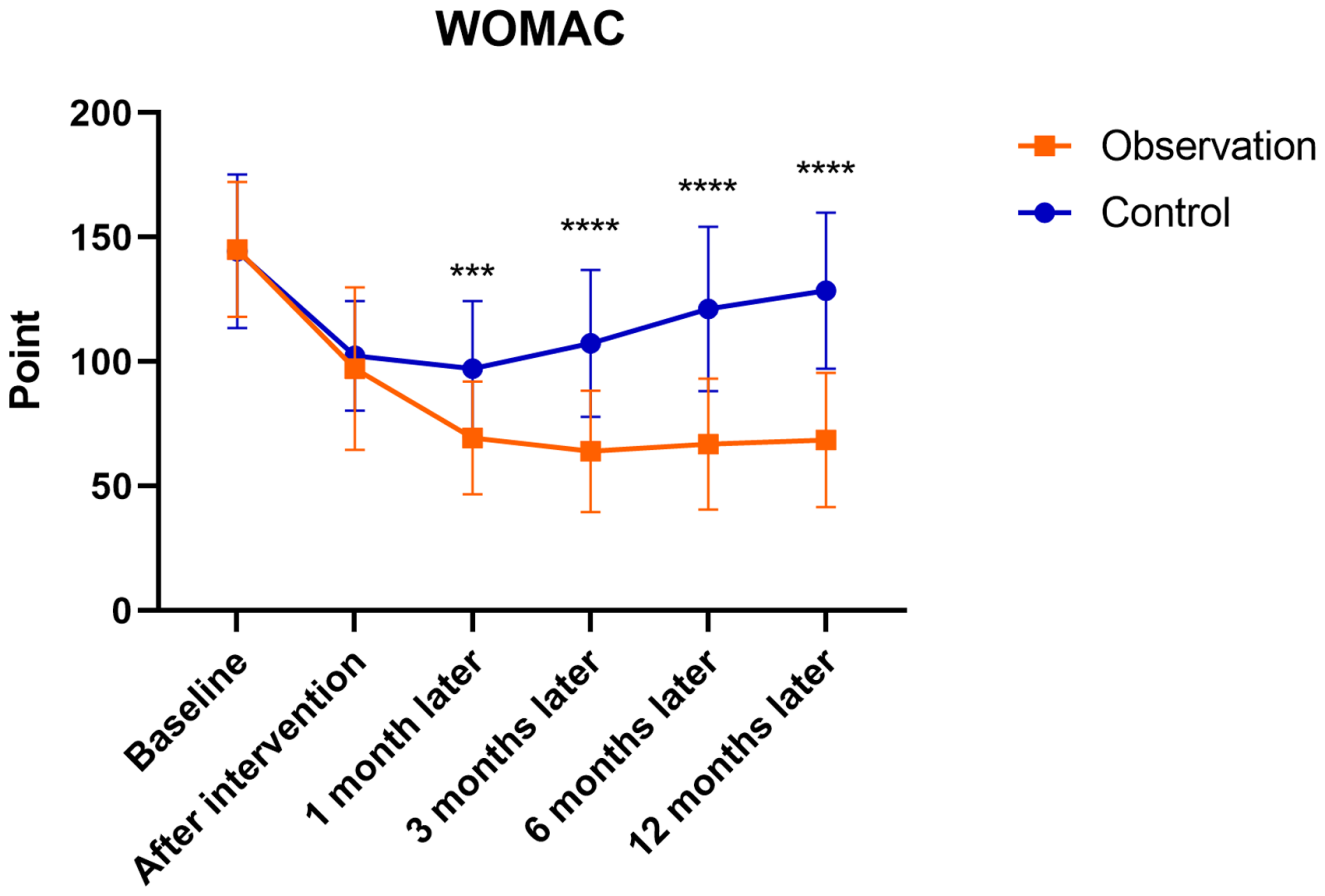
Mean WOMAC scores in the observation and control groups during the 12-month follow-up period. ^***^There was a statistically significant difference between the treatment groups at 1 month after intervention (*p* = 0.0002), ^****^From 3 to 12 months after intervention, there were significant differences between the treatment groups (*p* < 0.0001).

**Figure 7:**
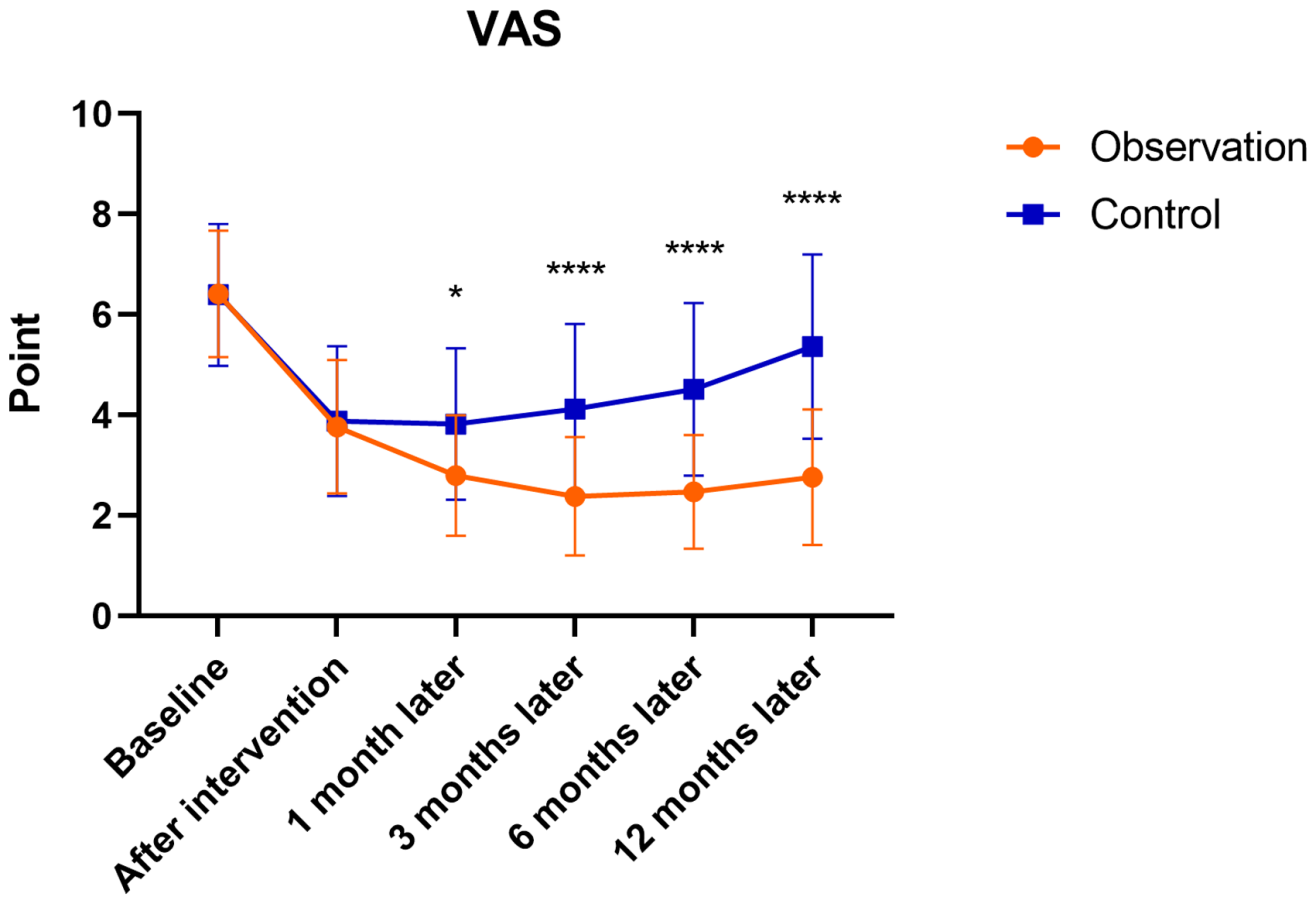
Mean VAS scores in the observation and control groups during the 12-month follow-up period. ^*^There was a statistically significant difference between the treatment groups at 1 month after intervention (*p* < 0.05), ^****^From 3 to 12 months after intervention, there were significant differences between the treatment groups (*p* < 0.0001).

There were significant differences in the WOMAC and VAS follow-up scores of patients in the observation group after the intervention to 12 months follow-up (*p* < 0.001). The scores were highest before the intervention and gradually decreased after the intervention, reaching the lowest point at 3 months after the intervention, with a slight increase at 6 months after the intervention. There were significant differences in the WOMAC and VAS follow-up scores in the control group after the intervention to 6 months follow-up compared to before treatment (*p* < 0.05). The scores were highest before the intervention, and gradually decreased after the intervention, which was the lowest at 1 month after the intervention, and then showed an upward trend to 12 months after the intervention. However, there were no statistically significant differences in WOMAC and VAS scores at 12 months after the intervention compared with those before the intervention (*p* > 0.05). There were no significant differences between the observation and control groups before or after the intervention. At 1, 3, 6, and 12 months after the intervention, the WOMAC and VAS scores in the observation group were significantly lower than those in the control group (*p* < 0.05; Figures 6, 7).

### 3.3. Security assessment

No life-threatening serious adverse events occurred during the study, and no adverse events required targeted medical measures, such as organ damage or severe infections. Some patients in this study experienced mild discomfort, including one patient in the observation group and two patients in the control group, who experienced transient worsening of joint pain after injection. This was considered to be due to the stimulation of the synovium during needle insertion for injection treatment. After resting and symptomatic treatment, the symptoms improved and resolved by the second day of treatment.

## 4. Discussion

The present study found that the levels of IL-6, IL-1β, TNF-α, IL-17A, and IL-10 in the synovial fluid of the observation group were significantly decreased after the intervention, while the levels of IL-8, IL-17F, and IL-4 showed a slight decrease and IL-5 showed a slight increase. WOMAC and VAS scores were significantly lower than those before the intervention. Indicating that PRP injection therapy can reduce the levels of certain inflammatory cytokines in the synovial fluid of KOA patients, reducing the secretion of inflammatory factors, alleviating inflammatory responses, relieving pain, improving joint function, and demonstrating superior therapeutic efficacy compared to the HA injection treatment group.

In this study, the concentrations of inflammatory cytokines, such as IL-6, IL-1β, TNF-α, IL-17A, IL-10, IL-8, IL-17F, and IL-4, in the synovial fluid of the observation group decreased compared to before the intervention. Indicating that PRP treatment reduces pain scores and improves clinical symptoms in KOA patients, thus achieving a pain-relieving effect. This was likely achieved by lowering the expression of inflammatory cytokines and clearing the pro-inflammatory factors. Knee osteoarthritis is the most common degenerative joint disease, and its pathophysiology is based on interactions between the bone, cartilage, and synovium. Synovial inflammation in KOA is characterized by mononuclear cell infiltration and the production of inflammatory mediators (25). Elevated levels of TNF-α, IL-1, and IL-6 have been observed in the synovial fluid, synovium, subchondral bone, and cartilage of patients with KOA. These cytokines can activate various inflammatory pathways and play a significant role in the pathogenesis of KOA, potentially exacerbating the severity of the disease by increasing the secretion of matrix metalloproteinases and prostaglandins and inhibiting proteoglycan and type II collagen synthesis, leading to joint swelling or cartilage damage (26). A reduction in the levels of these cytokines can disrupt the cycle of degenerative changes in the joint environment, slow disease progression, and restore the homeostasis of joint tissues (27). Multiple studies have indicated a correlation between the levels of inflammatory cytokines in the synovial fluid and OA severity (28, 29). Therefore, it is logical to assume that the clinical symptoms of KOA are directly related to cytokine levels. This study evaluated the changes in nine inflammatory cytokines relative to baseline levels following two treatment approaches, which objectively reflected the efficacy of PRP therapy for knee osteoarthritis.

Tumor necrosis factor-α and IL-1β are considered key cytokines in the pathogenesis of OA and are significantly elevated in OA patients. Additionally, these two cytokines are partially produced by OA articular chondrocytes, inducing the production of several inflammatory and catabolic factors (30). TNF-α and IL-1β are considered critical factors in the inflammatory cascade in KOA (31). These factors stimulate the production of proteases and prostaglandin E2 (PGE2), induce synovial cells and chondrocytes to produce other cytokines, such as IL-8 and IL-6, and stimulate their own production, accelerating joint tissue damage (32). IL-6 is a proinflammatory cytokine that downregulates the expression and synthesis of type II collagen (33). Research has found that IL-6 in the blood of early-stage KOA patients is associated with joint function, while TNF-α is associated with pain severity (34). TNF-α, as a cell signaling protein, plays a role in apoptosis and is an extremely effective pro-inflammatory cytokine that interacts with chondrocytes by binding to receptors on their surface. Animal experiments have shown that intra-articular injection of TNF in a rat model can lead to severe depletion of proteoglycans in the superficial layer of the cartilage (35). Furthermore, compared with a placebo, intra-articular injection of anti-tumor necrosis factor monoclonal antibodies in a rabbit OA model reduced the extent of cartilage damage (36). IL-17 is a potent osteoclastogenic cytokine whose receptors are expressed in various cell types, including synovial cells and chondrocytes, while IL-17A plays a role in driving synovial inflammation and joint destruction (37). IL-4, a cartilage protectant that inhibits the synthesis of IL-1β and TNF-β, is considered to have powerful therapeutic potential owing to its inhibitory effect on IL-1β, the main mediator of inflammation leading to cartilage degradation (38). *In vitro* studies reported that IL-4 and IL-10 can act at distinct levels within the TNF-α-dependent signaling cascade, reversing TNF-α-induced PGE2 release by OA synovial fibroblasts (39, 40). The decrease in IL-4 concentration observed in this study may be attributed to the negative feedback effect of reduced IL-1β and TNF-α concentrations. Mabey (41) found a positive correlation between IL-4 and IL-6 concentrations and the radiological severity of the disease. In this study, the observation showed a significant reduction in IL-6 and TNF-α levels after treatment, which could be attributed to the high concentration of growth factors present in the PRP, which reduced the synthesis of inflammatory factors and enzymes. Previous studies reported that PRP reduces the expression of IL-1β and TNF-β (42). This study also found that PRP lowered IL-6 levels, thereby enhancing its anti-inflammatory effects, which is one of the mechanisms through which PRP exerts its therapeutic effects.

Platelet-rich plasma exerts anti-inflammatory and analgesic effects and modifies the intra-articular environment in patients with KOA by stimulating the secretion of growth factors and reducing the production of pro-inflammatory cytokines (43). Inflammation and joint degradation represent the two biological mechanisms underlying KOA pathology and serve as primary targets of PRP therapy. Activation of the degradation process is typically a consequence of inflammation, which can affect all tissues comprising the joint. As OA progresses, synovial hyperplasia occurs, resulting in regression of fibrosis, increased vascular formation, and enhanced cell proliferation, migration, and invasion (44, 45). A recent proteomic study demonstrated an association between synovial proteins and their tissue-specific sources. In addition to the cartilage and synovium, the meniscus and infrapatellar fat pad also contribute to the protein composition of the synovial fluid of patients with KOA (46). Intra-articular PRP injections can alter the molecular composition of synovial fluid, which infiltrates joint tissues and regulates the microenvironment during this process (47). Moreover, PRP injection into the joint can dilute and replace the inflammatory synovial fluid with balanced cytokines and growth factors, which may serve as a foundation for pain improvement (48). Platelets can also mitigate the inflammatory response by regulating the infiltration and secretion of immune cells including TGF-b, PF4, NAP-2, RANTES, and MIP-1a among others (49). Furthermore, platelets interact with other cells of the immune system to modulate neutrophil degranulation and phagocytosis. Platelets reduce leukocyte infiltration in various acute and chronic inflammation models (50, 51). This study asserts that the focal point of PRP therapy for KOA lies in altering the balance between pro-inflammatory and anti-inflammatory cytokines, thereby reducing the secretion of certain inflammatory factors and eliminating inflammatory substances. Although the levels of inflammatory cytokines in the control group decreased to some extent, the reduction was not statistically significant. This indicated that the inflammatory environment within the synovial fluid of the knee joint did not show notable improvements. Although there was some improvement in joint function, the duration and efficacy of the treatment were not sustained. Conversely, in the observation group, WOMAC and VAS scores continued to decrease throughout the intervention and follow-up periods, reaching a plateau at approximately 12 months. The observation group experienced sustained pain relief and continuous improvement in joint function, illustrating that PRP injections not only significantly reduced patient pain and improved joint function, but also had longer-lasting effects, which can be attributed to the amelioration of the inflammatory environment within the knee joint cavity. PRP, a concentrated product of platelets derived from autologous plasma, possesses therapeutic potential owing to the abundant supply of various growth factors and anti-inflammatory cytokines synthesized within the platelets. These factors induce cell proliferation, migration, differentiation, angiogenesis, and extracellular matrix synthesis (52).

Herein, both patient groups had significant fluctuations in WOMAC and VAS scores over time. In the observation group, the WOMAC and VAS scores continued to decrease during the intervention and follow-up periods with statistically significant differences. At 6 and 12 months after the intervention, the WOMAC and VAS scores slightly increased compared to before treatment, indicating that the duration of PRP treatment’s effectiveness in KOA began to weaken at approximately 6 months but still significantly improved compared to before treatment. The WOMAC and VAS scores in the observation group at 1, 3, 6, and 12 months after the intervention were significantly lower than those in the control group. This indicates that PRP treatment for knee osteoarthritis can alleviate pain symptoms in the short term compared with the control group, and it has long-term effects on improving joint function and relieving pain. PRP improves the inflammatory environment in the synovial fluid of the knee joint, and has a unique effect on relieving knee joint pain and improving joint function. Chronic pain caused by KOA is caused by chronic inflammation resulting from chronic damage, which can continuously release inflammatory factors. These inflammatory factors can directly activate nociceptive neurons and induce central sensitization, thereby causing persistent pathological pain (53). The level of pain caused by knee osteoarthritis is closely related to the levels of inflammatory factors in the synovial fluid, such as IL-6, IL-1β, TNF-α, IL-8, etc. A study on the correlation between immune biomarkers and early- and late-stage MRI of KOA demonstrated a negative correlation between IL-6 levels and WOMAC scores. Early-stage pro-inflammatory biomarkers IL-6, IL-8, and TNF-α were also found to be associated with most MRI features (31). PRP not only directly promotes the release of anti-inflammatory cytokines and eliminates chronic inflammation but also exerts an anti-inflammatory effect by blocking the synthesis and release of pro-inflammatory factors, thus reducing pain. Multiple studies have confirmed that PRP inhibits inflammatory reactions, alleviates pain, and provides long-term relief. Its efficacy is comparable to that of corticosteroids, making it a potential alternative to knee joint surgery (54, 55). These findings confirmed that the anti-inflammatory effect of PRP was consistent with its ability to reduce pain and improve joint function. PRP treatment for knee osteoarthritis effectively lowers the concentrations of inflammatory factors IL-6, IL-1β, TNF α, IL-17A, and IL-10, significantly alleviating knee joint pain and enhancing joint functionality.

In conclusion, PRP therapy can decrease the concentration of inflammatory cytokines in the synovial fluid, improve the inflammatory environment of KOA joints, and reduce the inflammatory stimulation of knee joint tissues by inflammatory factors. This may be one of the mechanisms by which PRP is effective in the treatment of KOA. This study represents a singular investigation conducted at a specific center with a relatively limited sample size. The selection of biomarkers for knee osteoarthritis remains insufficiently comprehensive, as it does not encompass an examination of various significant components, such as the matrix metalloproteinase family, oligomeric cartilage matrix proteins, and microRNAs. Future multicenter, double-blind, randomized controlled trials with large sample sizes and extended follow-up periods to observe the long-term effects are required. Additional studies should be conducted to explore the mechanisms and signaling pathways involved in PRP therapy for KOA using advanced research designs, extensive proteomics, and big data methodologies.

## 5. Conclusion

Platelet plasma therapy can reduce the concentrations of inflammatory cytokines IL-6, IL-1β, TNF-α, IL-17A, and IL-10 in the synovial fluid of KOA patients, reduce the expression levels of IL-8, IL-17F, and IL-4, clear the pro-inflammatory factors, improve the inflammatory environment of the affected knee joint, and alleviate pain caused by inflammation. Thus, alleviating pain and improving knee function in patients with KOA.

## Data availability statement

The raw data supporting the conclusions of this article will be made available by the authors, without undue reservation.

## Ethics statement

The studies involving humans were approved by Ethics Committee of Henan Provincial People’s Hospital. The studies were conducted in accordance with the local legislation and institutional requirements. The participants provided their written informed consent to participate in this study.

## Author contributions

TL: Data curation, Writing – original draft. YL: Methodology, Writing – review & editing. WL: Data curation, Writing – review & editing. XW: Software, Writing – review & editing. QD: Formal Analysis, Writing – review & editing. JG: Formal Analysis, Writing – review & editing. YZ: Writing – review & editing, Methodology. WZ: Data curation, Writing – review & editing.
